# P28 Indications for central venous line access for treating infections and the appropriateness of the antimicrobial therapy: a clinical audit

**DOI:** 10.1093/jacamr/dlac004.027

**Published:** 2022-02-16

**Authors:** Kimberly Chong Shea Tin, Robert John Shorten

**Affiliations:** University of Manchester Medical School, Manchester, UK; Department of Microbiology, Lancashire Teaching Hospitals NHS Foundation Trust, Preston, UK; Centre for Clinical Microbiology, University College London, London, UK

## Abstract

**Introduction:**

Central venous access devices (CVADs) are used to deliver medications, including antibiotics. CVAD requests should be monitored closely due to the risks associated with insertion and the potential complications of venous thromboembolism and line-associated bloodstream infections. Inappropriate antibiotic use is associated with side effects, including healthcare-associated infections such as *Clostridioides difficile* infection and the emergence of antimicrobial resistance (AMR).

**Methods:**

An audit was performed to investigate the appropriateness of requests for CVAD insertion and the antimicrobials prescribed. In total, 52 patient records were analysed over a 1 month period.

**Results:**

There was good evidence of infection requiring IV antibiotics in 49/52 cases. Antibiotic selection was in line with guidelines in 71.4% of cases, and the vast majority of the exceptions had been discussed with a microbiologist. Good compliance was seen with guidelines for requesting CVAD access, but poor online documentation meant that it was not possible to confirm this in all cases.

**Conclusions:**

Despite some minor anomalies detected in this small patient size, there was an overall prominent demonstration of proficiency in clinical judgement by the central venous access team and microbiology team. The CVAD requests made by the clinical teams had definite indications and were predominantly appropriate. This highlights the importance of a multidisciplinary approach, particularly the microbiologist, when considering antimicrobial therapy via CVAD, in view of the risks associated with antibiotic misuse. In addition, patients undergoing CVAD placement should be closely monitored, and thus online record-keeping and documentation should be better improved to ensure quality healthcare practice and patient safety.

